# Immunotherapy with HDC/IL-2 may be clinically efficacious in acute myeloid leukemia of normal karyotype

**DOI:** 10.1080/21645515.2019.1636598

**Published:** 2019-07-24

**Authors:** Malin S. Nilsson, Alexander Hallner, Mats Brune, Staffan Nilsson, Fredrik B. Thorén, Anna Martner, Kristoffer Hellstrand

**Affiliations:** a TIMM Laboratory, Sahlgrenska Cancer Center, Department of Infectious Diseases, Institute of Biomedicine, Sahlgrenska Academy, University of Gothenburg, Gothenburg, Sweden; b Department of Internal Medicine and Clinical Nutrition, Institute of Medicine, Sahlgrenska Academy, University of Gothenburg, Gothenburg, Sweden; c Department of Mathematical Sciences, Chalmers University of Technology, Gothenburg, Sweden; d Department of Laboratory Medicine, Institute of Medicine, Sahlgrenska Academy, University of Gothenburg, Gothenburg, Sweden

**Keywords:** Acute myeloid leukemia, histamine dihydrochloride, IL-2, immunotherapy, relapse prevention, normal karyotype

## Abstract

Immunotherapy with histamine dihydrochloride and low-dose interleukin-2 (HDC/IL-2) reduces the risk of relapse in the post-chemotherapy phase of acute myeloid leukemia (AML). Here we report the results of exploratory analyses of the clinical efficacy of HDC/IL-2 in AML with focus on the impact of karyotype aberrations in leukemic cells. Post-hoc analyses of phase III trial data suggested that HDC/IL-2 is primarily beneficial for patients with AML of normal karyotype. These results may be helpful in the selection of patients who are suitable for therapy and in the design of future immunotherapy protocols aiming at further defining the mechanism of relapse prevention by HDC/IL-2.

Acute myeloid leukemia (AML) is characterized by rapid clonal expansion of immature myeloid cells in bone marrow. After initial rounds of chemotherapy (induction), a majority of patients attain complete remission (CR), defined as less than 5% leukemic blasts in the bone marrow along with the return of non-malignant hematopoiesis. Ensuing courses of chemotherapy (consolidation) aim at eliminating residual leukemia. However, approximately 60–70% of adult patients in first CR (CR1) experience relapse of AML, ^1^ mostly within 2–3 years, with dismal prognosis for long-term survival. The absence of strategies to prevent relapse in the post-chemotherapy phase in non-transplanted patients is a significant reason why the rate of 5-year survival in adult AML is in the range of 25–30%.^1^

AML cells invariably carry somatic genetic aberrations that may be grouped by chromosomal morphology into AML of aberrant or normal karyotype. In approximately 55% of human adult AML, the leukemic cells thus carry structurally or numerically aberrant chromosomes, including deletion or multiplication of chromosomes or chromosome sections [*e.g.* −5, −7, +8, +21, +22, del(5q), del(7q), del(9q)] or inverted/translocated chromosome arms or segments [*e.g*. t(8;21), t(9;11), t(15;17), inv(16)]. Normal karyotype AML, where the chromosomes of leukemic cells are morphologically and numerically intact, comprises mutations in *e.g. NPM1, FLT3-ITD, CEBPA, DNMT3A, IDH1* and *IDH2* and constitutes approximately 45% of all adult AML. The mere classification of AML into aberrant or normal karyotype does not provide major prognostic information. Instead, there are entities within these groups of gene aberrations that herald variable prognosis. Relapse risk and/or survival are thus favorably impacted by t(8;21), t(15;17) and inv(16) translocations along with *NPM1* or *CEBPA* mutations, whereas somatic mutations in, *e.g., FLT3-ITD* or deletions of chromosomes 5 or 7 are associated with poorer prognosis.^2,3^

Aspects of lymphocyte function and phenotype have been reported to impact on the risk of relapse in the post-consolidation phase of AML, and several immunotherapies have been developed aiming at reducing the relapse risk.^4^ These include administration of antibodies against leukemia-associated antigens, treatment with immunostimulatory cytokines, adoptive transfer of anti-leukemic lymphocytes and vaccine strategies using dendritic cells carrying leukemic antigens or fused with autologous leukemic cells.^4,5^ Of these, only immunotherapy with histamine dihydrochloride and low-dose interleukin-2 (HDC/IL-2) has proven efficacious in terms of relapse prevention in a randomized phase III setting^6^ (reviewed in)^4^. Treatment with HDC/IL-2 aims at targeting the formation of immunosuppressive reactive oxygen species produced by the NOX2 enzyme of myeloid cells (HDC component), while concomitantly activating and expanding populations of natural killer (NK) cells and T cells (IL-2 component). These components act in synergy to promote NK and T cell function and viability *in vitro*, and also synergize to reduce or inhibit tumor growth in animal models *in vivo*^7–9^ (reviewed in)^4^.

Here we report the results of exploratory analyses of the clinical efficacy of HDC/IL-2 in AML with focus on the impact of genetic aberrations in leukemic cells. Our data suggest that HDC/IL-2 is primarily beneficial for patients with AML of normal karyotype. The analyses presented are founded on databases of a phase III study^6^ and a phase IV trial^10^ using HDC/IL-2 for relapse prevention in AML. In the phase III trial, 320 AML patients in CR who had completed consolidation chemotherapy were randomized to treatment with HDC/IL-2 (n = 160) or standard of care (no treatment; n = 160). Patients were median 57 (18–84) years at inclusion. Two-hundred sixty-one patients were in their first CR (CR1), and 59 in their second or subsequent CR (CR>1). Patients were recruited at 100 centers in Europe, USA, Australia and New Zealand. Details of study procedures for random assignment, exclusion criteria, dosing, treatment schedules and toxicity are accounted for in a previous report.^6^ In brief, patients in the treatment arm received up to ten cycles of HDC (0.5mg s.c. bid) and low-dose IL-2 (16,400U/kg s.c bid) for 21 days followed by 21 days of rest. Beginning in cycle 4, the rest periods were extended to 42 days. Treatment continued for up to 18 months, and all surviving patients were followed for at least 36 months. The primary trial endpoint was leukemia-free survival (LFS, defined as the time from randomization to relapse or death from any cause). Secondary endpoints included overall survival (OS) and efficacy in terms of LFS and OS in subgroups such as CR status (CR1 or CR>1 at randomization), age (<60 or ≥60 years) and baseline characteristics including genetic aberrations in leukemic cells.

The phase IV trial (Re:Mission Trial;^10^) was a single-armed multicenter phase IV study that enrolled 84 patients (age 18–79, median 61) with AML in CR1 who were not eligible for allogeneic transplantation. All patients received HDC/IL-2 using a schedule and dosing identical to that employed in the phase III trial. All surviving patients were followed for at least 24 months after enrollment. The endpoints included assessment of the quantitative and qualitative pharmacodynamic properties of HDC/IL-2 on immune populations, including T and NK cell phenotypes, as analyzed before and after treatment cycles. Forty-four of the patients in the phase IV trial harbored AML cells of normal karyotype (age 28–74, median 60). Fifteen of the normal karyotype patients (age 28–74, median 60) carried leukemic cells with *NPM1* mutations (out of 36 analyzed; 42%), whereas four patients (age 53–64, median 64) carried *FLT3-ITD* mutations (out of 38 analyzed; 11%). Three of the latter patients carried mutations in both *NPM1* and *FLT3-ITD*. Further patient characteristics including previous induction and consolidation therapies and risk group distribution are accounted for elsewhere.^10,11^ The phase III and phase IV trials were approved by the Ethical Committees of each participating institution and all patients gave written informed consent before enrollment. The logrank test was applied to analyze the impact of treatment with HDC/IL-2 on LFS and OS in subgroups of patients harboring leukemic cells with normal or aberrant karyotype as defined at diagnosis.

The potential efficacy of HDC/IL-2 was assessed in CR1 patients with normal or aberrant karyotype AML who participated in the randomized phase III trial. The karyotypic features of leukemic cells were unknown in 36 of the 261 patients in CR1. Thus, data obtained from 225 CR1 patients with known karyotype were available for analysis of clinical outcome (LFS and OS). There was no discernable treatment effect on outcome (LFS or OS) among patients in CR1 with aberrant karyotype. By contrast, HDC/IL-2-treated patients with normal karyotype AML displayed improved LFS over control patients with a trend towards improved OS (Figure 1a and b). HDC/IL-2 was not significantly efficacious in older patients with aberrant or normal karyotype. Younger patients (<60 years) with normal karyotype AML showed significantly improved LFS and OS vs. control patients (Figure 1c and d). These results imply that the clinical benefit of HDC/IL-2 in AML is pronounced in patients harboring leukemic cells of normal karyotype.

**Figure 1.**
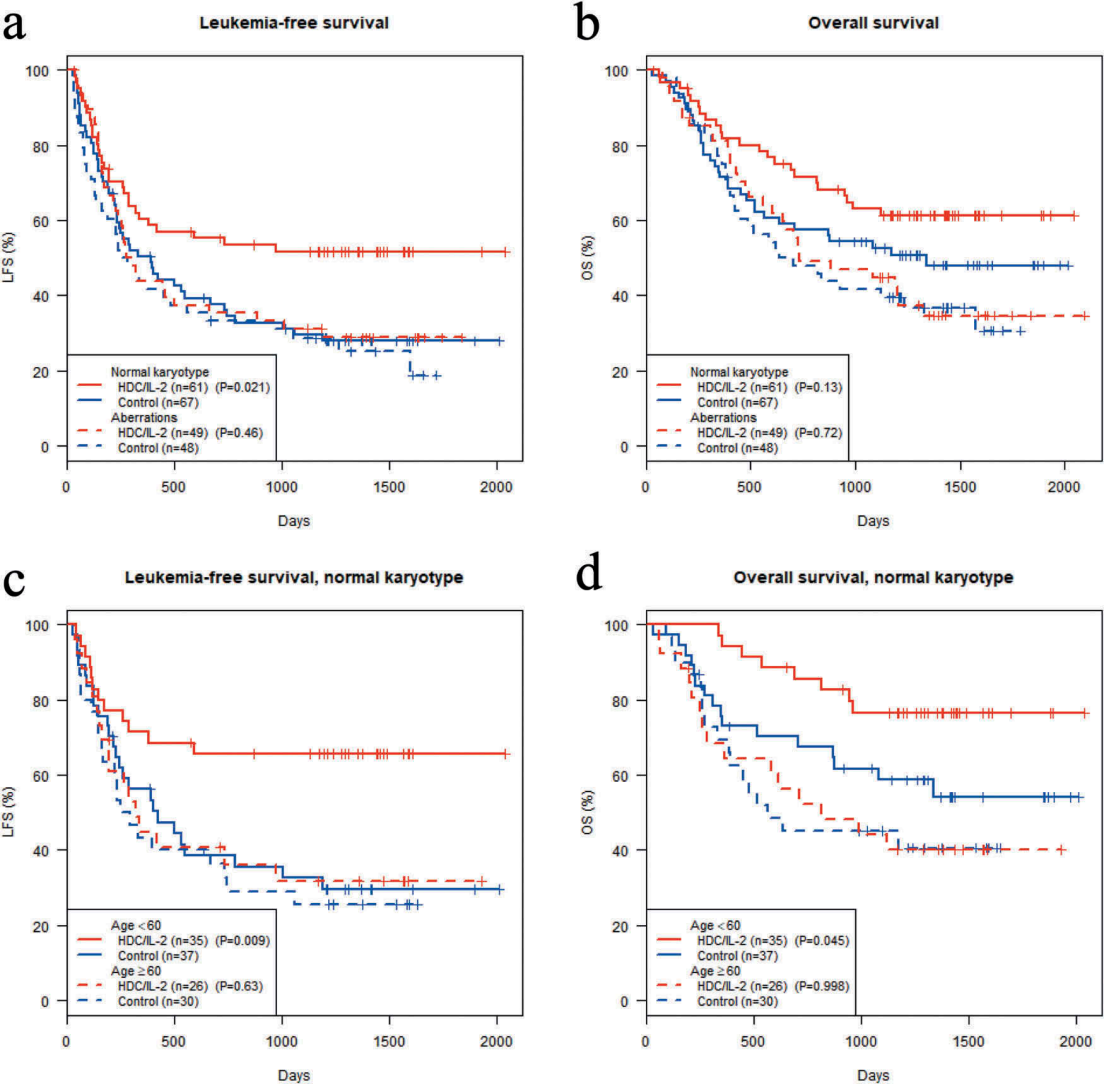
**Clinical efficacy of HDC/IL-2 in patients with aberrant or normal karyotype AML**. Analysis of the impact of HDC/IL-2 treatment on leukemia-free survival (LFS) and overall survival (OS) in a phase III trial. The Kaplan-Meier plots show LFS (a) or OS (b) in patients in CR1 with known karyotype (n = 225). Figures (c) and (d) show corresponding results in patients with normal karyotype AML who were below or above 60 years old at random assignment. The logrank test was employed for statistical analysis.

Mutations of *NPM1* or *FLT3-ITD* are the most prevalent mutations within the group of normal karyotype AML, where mutated *NPM1* comprises approximately 40–50% of cases and *FLT3-ITD* approximately 30–40%.^12^ During the course of the phase III trial, analyses of *NPM1* and *FLT3-ITD* were not part of clinical practice. However, in the ensuing single-arm phase IV trial using HDC/IL2 this information was available, and we thus analyzed outcome of patients with the two dominant mutations in normal karyotype AML. The results did not support efficacy of HDC/IL-2 in patients harboring mutated *FLT3-ITD* as 4/4 patients relapsed within 200 days. The outcome was, however, as expected, more favorable in patients diagnosed with *NPM1*-mutated AML devoid of karyotype aberrations or *FLT3-ITD* mutations, where 67% of patients (n = 14) were alive and relapse-free at 2 years.

In conclusion, the phase III trial results point to the possibility that HDC/IL-2 is clinically efficacious in AML patients with leukemic cells of normal but not aberrant karyotype, and that this aspect of clinical efficacy is pronounced in younger patients. The magnitude of efficacy of HDC/IL-2 in AML patients with normal karyotype, in particular in patients <60 years, suggests that the treatment effect is present within the groups of *NPM1*- and/or *FLT3-ITD*-mutated AML, as these mutations account for >75% of all normal karyotype AML.^12^ The results of the phase IV trial argue against the possibility that HDC/IL-2 is beneficial in patients with normal karyotype AML carrying *FLT3-ITD*-mutations, thus raising the possibility that HDC/IL-2 may be primarily efficacious in *NPM1*-mutated AML. The exploratory nature of these results should be emphasized, and controlled trials are warranted to determine the potential benefit of HDC/IL-2 in defined genetic subgroups of normal karyotype AML.
